# Global processing provides malignancy evidence complementary to the information captured by humans or machines following detailed mammogram inspection

**DOI:** 10.1038/s41598-021-99582-5

**Published:** 2021-10-11

**Authors:** Ziba Gandomkar, Somphone Siviengphanom, Ernest U. Ekpo, Mo’ayyad Suleiman, Seyedamir Tavakoli Taba‬, Tong Li, Dong Xu, Karla K. Evans, Sarah J. Lewis, Jeremy M. Wolfe, Patrick C. Brennan

**Affiliations:** 1 Discipline of Medical Imaging Sciences, Faculty of Medicine and Health, University of Sydney, 512/Block M, Cumberland Campus, Sydney, NSW 2006 Australia; 2 School of Electrical and Information Engineering, Faculty of Engineering, University of Sydney, Sydney, NSW 2006 Australia; 3 Department of Psychology, University of York, York, UK; 4 Harvard Medical School, Boston, MA USA; 5 Brigham and Women’s Hospital, Boston, MA USA

**Keywords:** Psychology, Human behaviour, Cancer, Breast cancer

## Abstract

The information captured by the gist signal, which refers to radiologists’ first impression arising from an initial global image processing, is poorly understood. We examined whether the gist signal can provide complementary information to data captured by radiologists (experiment 1), or computer algorithms (experiment 2) based on detailed mammogram inspection. In the first experiment, 19 radiologists assessed a case set twice, once based on a half-second image presentation (i.e., gist signal) and once in the usual viewing condition. Their performances in two viewing conditions were compared using repeated measure correlation (rm-corr). The cancer cases (19 cases × 19 readers) exhibited non-significant trend with rm-corr = 0.012 (*p* = 0.82, CI: −0.09, 0.12). For normal cases (41 cases × 19 readers), a weak correlation of rm-corr = 0.238 (*p* < 0.001, CI: 0.17, 0.30) was found. In the second experiment, we combined the abnormality score from a state-of-the-art deep learning-based tool (DL) with the radiological gist signal using a support vector machine (SVM). To obtain the gist signal, 53 radiologists assessed images based on half-second image presentation. The SVM performance for each radiologist and an average reader, whose gist responses were the mean abnormality scores given by all 53 readers to each image was assessed using leave-one-out cross-validation. For the average reader, the AUC for gist, DL, and the SVM, were 0.76 (CI: 0.62–0.86), 0.79 (CI: 0.63–0.89), and 0.88 (CI: 0.79–0.94). For all readers with a gist AUC significantly better than chance-level, the SVM outperformed DL. The gist signal provided malignancy evidence with no or weak associations with the information captured by humans in normal radiologic reporting, which involves detailed mammogram inspection. Adding gist signal to a state-of-the-art deep learning-based tool improved its performance for the breast cancer detection.

## Introduction

Radiologists can detect the “gist of the abnormal” in mammograms based on a half second glimpse of the image^1^. That is, with only a brief glimpse, expert observers can categorize images as normal or abnormal at above chance levels. In the context of medical image perception, the radiologist’s first impression about the presence of the abnormality in an image, is called the gist signal or gist response. There are two accounts of gist in medical images and, indeed, there are probably two types of “gist”. One account assumes that the gist signal is based on rapid processing that can guide the eyes to the location of the target. The Kundel et al. “holistic processing model” assumes a gist signal with a localized source^2^. Alternatively, recent studies^3–5^ suggest another type of gist signal, which arises from global image properties and does not rely on the presence of a localizable lesion. It is hypothesized that such a signal is associated with the textural changes in the breasts of women at higher risk of current or future breast cancer^6^. In support of the existence of a global signal, it has been shown that radiologists can categorize images as normal or abnormal even in prior negative mammograms of women, who would eventually develop a breast cancer, but who have no overt signs of cancer in the images used in the study^7,8^. Additionally, it has been reported that the gist of the abnormal is present in the normal breast, contralateral to a malignancy^9^. Finally, in our previous study, mammograms containing cancer, lesion size^7^ or their difficulty in usual viewing condition^10^ was not associated with the strength of the gist signal.

In the usual viewing and reporting conditions, radiologists assess suspicious locations by prolonged foveal verifications^11^, re-fixate multiple times on the reported areas, move back and forth between two locations, possibly to compare them, and show frequent laminar movements (i.e. detailed inspection of an area with multiple consecutive fixations in a same area) on the suspicious locations^12^. In this process, the gist signal is sometimes overruled following further image inspection. If the gist signal relies on the global image statistics rather than a localized source within the image, it could be providing complementary information to the data acquired by radiologists or by a computer algorithm based on localized inspection of image. Therefore, this gist information could be useful for improving cancer detection and risk prediction. This paper focus on investigating the potentials of the gist signal for the cancer detection.

To investigate the nature of gist information, this study investigated if the gist responses and performances of the same radiologists in the gist experiment viewing condition are correlated with their ratings and performances in the usual presentation and reporting condition. An absence of or a weak correlation between radiologists’ assessments in the gist experiments and usual reporting conditions would suggest that separate but complementary information is being employed to decide whether a cancer is present. As our data showed an absence of correlation for the cancer cases and a weak correlation for normal cases, we also explored whether combining the radiologists’ decision in the usual presentation and reporting condition with the gist signal, could result in an improvement in the performance of the radiologist in identifying breast cancer. We also investigated whether it is possible to combine the gist signal with a state-of-the-art deep learning-based computer-aided detection tool^13^ to improve its performance. Like radiological scan paths, the tool relies on a deep filter for sweeping the image. Therefore, we hypothesize that adding the gist information could improve the performance of the tool.

## Results

### Experiment 1: Gist versus normal viewing response

In the first experiment, we aimed at exploring if the radiologist’s decision about a case in usual viewing and reporting condition is related to their first impression about presence of the abnormality, as measured by the gist response. To explore this relationship, 19 breast radiologists were asked to assess a data set of 60 cases (19 cancer cases, 41 normal cases) twice, once in a flashing mode and once in usual viewing condition. In the flashing mode, the images were presented for a half-second and radiologists were provided an abnormality score ranging from 0 (confident normal) to 100 (confident abnormal) to the image. In the flashing mode, both right and left crania-caudal mammograms were presented, and the maximum abnormality score was considered as the gist response. In the usual presentation condition, radiologist rated a case from 1 to 5 the Royal Australian and New Zealand College of Radiologists (RANZCR) rating system. It classifies the findings into no significant abnormality (1), benign (2), equivocal (3), suspicious (4), and malignant (5). Therefore, in the first experiment for each case and each radiologist, two scores were available, one from the gist experiment and one from the usual presentation and reporting condition.

#### Overall performance in two experiments

For each viewing condition, the receiver operating characteristics (ROC) curves for each reader was generated. The radiologists’ performances in the two viewing conditions, as measured by the area under the area under theses ROC curve (AUC), were not significantly correlated (Spearman correlation = 0.183, *p *value = 0.45). The average AUCs in the usual viewing condition and the gist experiment were 0.895 ± 0.047 and 0.771 ± 0.056. The AUCs from the gist experiment ranged from 0.668 to 0.908 and differed significantly (*p* < 0.001) from 0.5, i.e., the AUC of the chance level.

#### The association between the gist responses versus the ratings in usual reporting and viewing conditions

**Figure 1:**
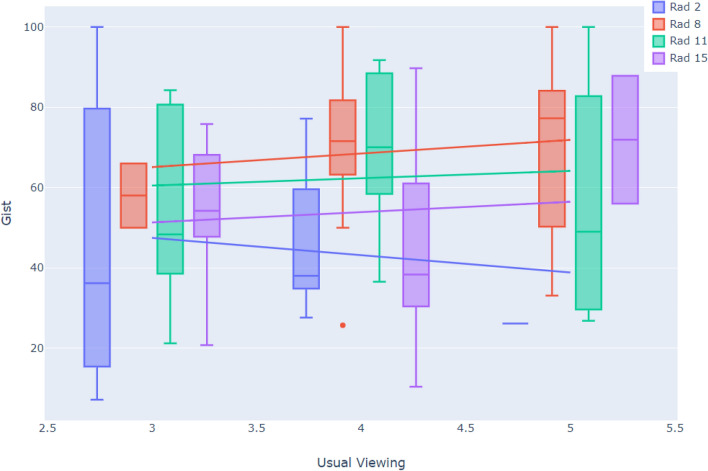
The gist responses and ratings in the usual viewing condition for cancer cases from four radiologists. The R2 for the fitted trendlines were less than 0.02 for all readers.

For each radiologist, a Spearman’s correlation was used to assess the association between the gist responses versus the ratings in usual reporting and viewing conditions for normal and cancer cases. The average correlations were 0.24 ± 0.1 and 0.05 ± 0.24 for normal and cancer cases, respectively. We analyzed two categories separately as both gist scores and RANZCR ratings are predictive of the case category (normal or cancer) and pooling both categories in the correlation analysis would result in two clusters, one corresponding to the normal and one corresponding to the cancer cases and hence pooled data would exhibit a strong correlation. However, our question was whether within each one of these clusters an association between gist scores and RANZCR ratings was present. The correlation values suggest no trend to weak positive trend for normal cases. Only for five radiologists a *p*-value < 0.05 for the correlation coefficient was achieved. On average, no trend for the cancer cases was observed. As an example, four radiologists were randomly selected and the distribution of their gist responses in various RANZCR rating categories (based on their own ratings in the usual viewing condition) for cancer cases is shown in Fig. 1. As shown, the data exhibited lack of any trend.

As radiologists were assessing the identical set of images, pooling the points from all radiologists and calculating the correlation between two viewing modes, would result in a dependency between samples. We used the repeated measure correlation (rm-corr), where the assumption of independence can be violated^14,15^. The rm-corr shows the associations between two scores, shared among radiologists. The Degree of Freedom (DOF) for the rm-corr^15^ was N × (k−1)^−1^, where N and k show number of radiologists and number of measurements per radiologists, respectively. The rm-corr for the cancer cases exhibited no trend with a value of 0.012 (*p* = 0.82, DOF: 341, 95% CI: −0.09, 0.12). For normal cases, the analysis led to a significant but weak correlation with an rm-corr = 0.238 (*p* < 0.001, DOF: 759, 95% CI: 0.27, 0.37).

The mixed linear regression model for the normal and cancer cases was built with gist responses as its inputs. Similar to the repeated measure correlation, the mixed linear regression model^16^ can be used when there is dependency among samples. Mammographic density was also included in both models. For cancer cases, the size of cancer and its type (calcification or not) were also fed into the model. The results are presented in Table 1. For cancer cases, the presence of mass associated with a higher RANZCR rating compared to cases with malignant calcifications. Also, as expected, a higher breast density will lead to a drop in the RANZCR rating. In other words, an average radiologist assigned a lower RANZCR rating (was more uncertain), when a cancer case was dense. As expected, a larger lesion size led to an increase in the RANZCR rating while gist responses failed to show any association with the RANZCR rating of the cancer cases. For normal cases, density led to a non-significant p-value while each level of increase in the gist signal was associated with an increase of 0.017 in the RANZCR rating. This positive association implies that the gist signal could contribute to the radiologists’ false-positive decisions.

**Table 1:** Mixed linear regression models.

	Coef	SE	*p*-value	CI [0.025 0.975]
**Cancer cases**				
Intercept	3.692	0.132	***p* < 0.001**	3.433–3.951
Mass: Calcification	0.193	0.068	**0.005**	0.059–0.326
Density	−0.052	0.026	**0.043**	−0.102–0.002
Size	0.011	0.005	**0.022**	0.002–0.021
Gist	0.000	0.001	0.755	−0.002–0.003
**Normal cases**				
Intercept	0.548	0.172	**0.001**	0.211–0.885
Density	−0.123	0.064	0.054	−0.248–0.002
Gist	0.017	0.002	*p* < 0.001	0.012–0.022

The model generated separately for normal and abnormal cases. Note that although larger sample size in Normal category could result in more precision and increase power, a coefficient of zero for the “Gist” response suggests that it is not a strong predictor of ratings in the usual presentation for the Cancer Cases.

Bold values showed the significant *p*-values

#### Combining gist with the assessment in the usual viewing condition

**Figure 2:**
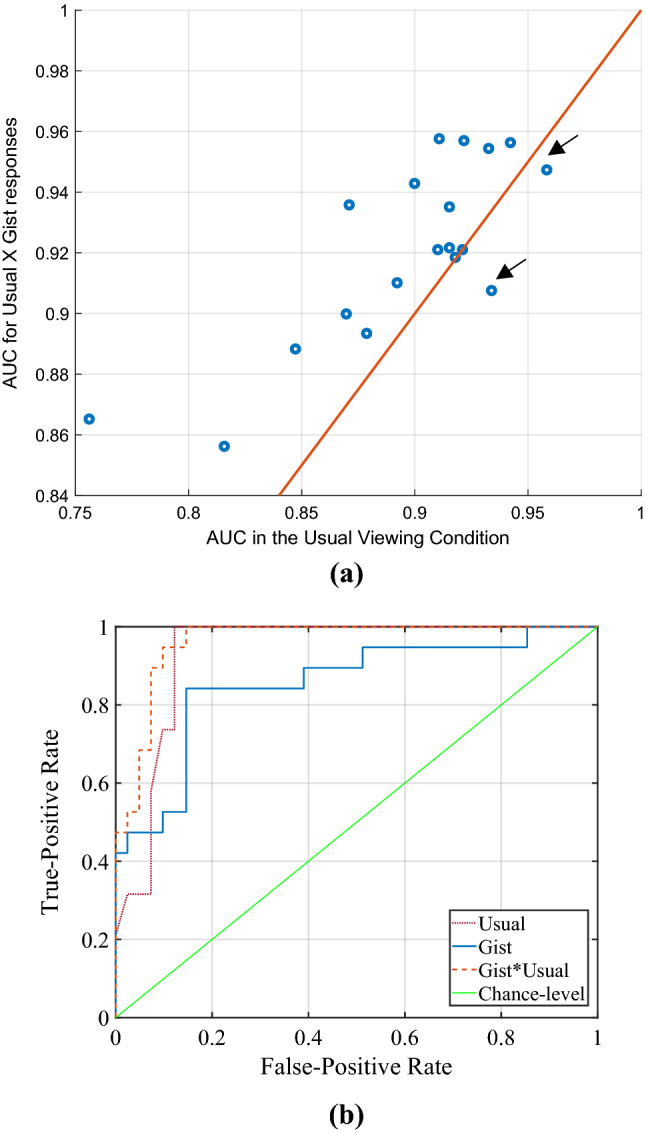
(**a**) The AUC value of the readers when the ratings in the usual viewing condition and gist responses were combined versus the AUC values of the readers in the usual viewing condition. The x = y line is also indicated (red line). As shown, the multiplication by the gist response resulted in the deterioration of the performance only in two of the readers (indicated by arrow). (**b**) The ROC curves of the average reader in the usual viewing and reporting condition, gist experiments, and when two scores were combined. The AUC values are 0.93 (0.86–0.98), 0.85 (CI: 0.68–0.93), and 0.96 (CI: 0.91- 0.99) respectively.

Considering the lack of correlation between two metrics of malignancy, we explored if combining them would improve the performance. To combine these two scores, we multiplied the rating in the usual viewing condition by the gist responses. Multiplying is a common approach for combing two different classifiers’ outcomes. The AUC value of the readers when two scores were combined versus the AUC values of the readers in the usual viewing condition is shown in Fig. 2a. The paired Wilcoxon signed-rank test indicated that the AUC after combining is significantly higher than the AUC of readers in the usual viewing condition (*p* = 0.0014). On average, the AUC increase 2.98% ± 3.72% and the percentage increase in AUC after the multiplication ranged from −2.82 to 14.4% (median of 2.17%).

We also produced an average reader, whose gist responses and ratings in the usual reporting condition was an average of all scores given by 19 radiologists to each case. By doing so, the noisiness of the gist signal was cancelled out. The AUC values of the average reader on his own in the usual viewing condition and the gist experiments were 0.93 (0.86–0.98) and 0.85 (CI: 0.68–0.93), respectively. When two scores were combined an AUC of 0.96 (CI: 0.91–0.99) was yielded. The ROC curves for these three conditions are shown in Fig. 2b.

#### Reader characteristics and their performances in two viewing modes

We also collected reader characteristics related to their experience and workload. The Mann–Whitney U test showed that none of the reader characteristics resulted in a significant difference between their performance based on the gist signal as measured by the AUC. However, grouping based on number of hours per week currently spent reading mammograms (*p* = 0.028), number of years they have been reading mammograms (*p* = 0.041), and number of cases read per week (*p* = 0.044) led to as significant difference in the readers’ AUCs during usual viewing and reporting condition.

### Experiment 2: Gist to complement to a deep learning model’s diagnosis

In the second experiment, we collected the gist responses of 53 radiologists for a case set of mammograms (20 cancer, 40 benign, and 20 normal). We generated the gist responses of the average observer, whose responses were defined as the average of the gist responses from all 53 radiologists for each case. The gist responses and an abnormality score from a state-of-the-art deep learning-based tool^13^ were fed into a support vector machine (SVM) to output the final abnormality score for the case and investigated the usefulness of combining the gist with the model’s output. The performance was evaluated using the leave-one-out cross-validation. Further description about the tool is provided in the "Materials and methods" section.

#### Performance of the gist-sensitive deep learning model

**Figure 3:**
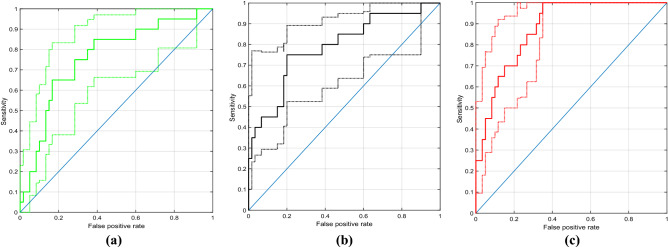
The ROC curves for the average reader (**a**), the model on its own (**b**), and when the gist signal is aggregated with the models’ output using an SVM for the average reader. The confidence intervals (dashed lines) were calculated using bootstrap. The number of bootstrap replicas was set to 100.

Figure 3 shows the ROC curve of the deep learning tool on its own, gist response of the average reader, and the SVM combining the gist response of the average reader with the model’s output. The confidence intervals are also shown with dashed lines. The AUC values were 0.76 (95% CI: 0.62–0.86), 0.79(95% CI: 0.63–0.89) and 0.88 (95% CI: 0.79–0.94) for average reader, the model on its own, and the gist signal combined with the models’ output using an SVM for the average reader respectively.

**Figure 4:**
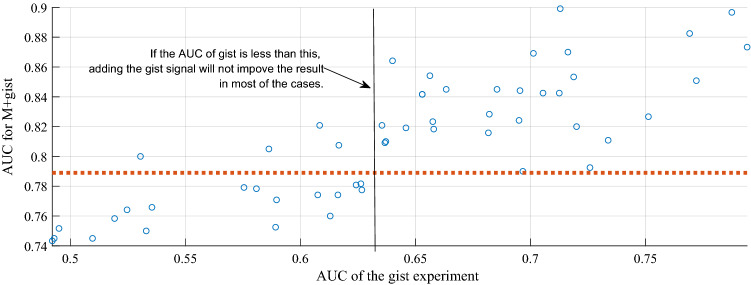
The AUC of the SVMs personalized for each radiologist versus their AUCs in the gist experiment. The dashed line shows the AUC of the model on its own. As shown in the figure, for most of the readers with an AUC above 0.635, an improvement in the model’s performance was observed after aggregating the gist signal with the deep learning’s abnormality score.

We also personalized the SVM for each radiologist and assessed the effect of combining two inputs using leave-one-out cross-validation. Out of 53 recruited radiologists, adding the gist responses of 34 radiologists improved the AUC of the model compared to when it works on its own. On average the AUC of the model improved by 2.85% ± 5.31%. The change in the performance of the model ranged from −5.77 to 13.96%, when the individual gist responses were added to the model. Figure 4 shows the AUC of the SVMs personalized for each radiologist versus their AUCs in the gist experiment. As shown, for all radiologists with a gist AUC of 0.635 or above, an improvement in the model’s performance was noted. This is an interesting observation, as such threshold is very close the minimum required AUC in the gist experiment to get an AUC with lower bound of 0.5 (i.e., chance-level) or more. Using Hanley and McNeil’s method^17^ to calculate the lower bound of AUC values, the radiologists’ gist AUC of 0.65 or more would be required to have lower bound at above chance-level. Therefore, the gist responses of all readers, whose performances in the gist experiment were significantly better than chance-level (i.e., equivalent of having a lower bound of 0.5 or more), could have improved the deep learning-based breast cancer detection tool.

## Discussion

In this paper, we investigated whether gist responses can identify mammograms with cancer based on information other than that employed during the localized image inspection, by 2 methods: scored by radiologists or a deep learning tool. The results obtained in the first experiment suggested that the gist response of radiologists in cancer cases was not a predictor of malignancy ratings in viewing conditions similar to the clinical environment. However, cases with lower breast density and larger lesion size were found to be associated with the higher ratings. For normal cases, higher gist responses were associated with the higher rating; however, the correlation was weak. Moreover, the performances in two viewing modes (gist vs usual viewing condition), were not correlated. A previous study showed that the case difficulty in usual viewing condition was not related to strength of the gist signal. The finding of this previous study and the results presented here are supportive of the hypothesis that the gist signal provide information about the presence of cancer using mechanisms other than those used in the detailed image inspection and when compared with the usual viewing condition, albeit, different skill sets may be needed for perceiving the gist of the abnormal.

The results of the second experiment suggested the possibility of using the gist response to improve the performance of a state-of-the-art deep learning model^13^ for detecting breast cancer. The gist response can be considered as an input to control the operating point of the deep learning model. In this sense, the proposed model could be identified as a “gist-sensitive” deep learning model. For each ROC curve, strict, moderate, or lax operating point can be selected. The strict operating points led to low false-positive rate at the expense of low sensitivity while the lax operating point results in a high sensitivity at the cost relatively large number of false-positives. The moderate operating point usually represents a knee point in the curve, where a balanced trade-off between the false-positive rate and sensitivity is obtained. Here, the SVM was trained to select the best cutoff point for the abnormality score based on the gist responses.

The patch classifier of the deep learning model^13^ essentially sweeps the input mammogram in both directions horizontally and vertically, encoding local malignancy features of masses and calcifications. It should be noted that adding layers on the top of patch classifier (the max-pooling and fully connected layers), aggregates the information from the entire input mammogram and makes the whole image classifier translational invariant. Therefore, in predicting the image-level label, presence of the lesion is a key factor for predicting the image-level classification and it does not matter where the lesion (i.e., localized source of malignancy feature) is. As an example, no matter a detected malignant lesion is in the upper-outer quadrant or lower inner quadrant, the image-level label is malignant. To further explain the decision making process of the model, Shen et al.^13^ used saliency maps, created using the guided back-propagation approach^18^, to illustrates which areas on an input mammogram is considered to be responsible for the cancer prediction by the whole image classifier. The analysis of the saliency maps showed that the identified area on the saliency map is in or close to the malignant regions. It was concluded that the whole image classifier was able to correctly locate the cancerous regions on which its decision was based^13^. In this sense, the saliency map analysis supports that the signal on which the deep learning model relies to make a diagnosis has a localized source although the network is not sensitive to where (in a mammogram) a malignant lesion is.

Shen et al.^13^ trained the model in two steps. First, the patch classifier was trained using annotated data and then they used an end-to-end training approach to further fine-tuning the model using datasets without annotations. It might be argued that the “end-to-end” training of the whole deep learning model and further updating the weights of the layers corresponding to the patch classifier might have resulted in capturing global image features. This is to some extent true, as the end-to-end training would help model to learn the context of lesions. However, the two-stage training strategy^13^ used to convert the patch classifier to the whole image classifier will not result in a significant change in the weight of the patch classifier layers. In the two-stage training strategy, first only the newly added top layers (i.e., layers added to top of the patch classifier) was trained with a learning rate of 10^−4^ for 30 epochs and then all layers were trained with a smaller learning rate of 10^−5^ for 20 epochs. The learning rate controls the “step”, which was made along the gradient to update the weight and number of epochs shows number of times the weight was changed. The smaller learning rate with fewer epochs implies that tiny steps were taken. Therefore, the second stage of the training ensures further fine-tuning of the patch classifier layers without dramatic change in the weights of the patch classifier layers as and training the patch classifier layers with a large learning rate may destroy relevant malignancy features already learned by these layers. Therefore, despite end-to-end training of the model and learning some contextual features, the bottom layers (i.e., the layers corresponding to the patch classifier) of the network still locate the malignant lesions and the top layers rely on this information to make the final image-level classification. As stated, this was also supported by the saliency map analysis.

One way to use the signal in future would be asking readers to first assess the images in flashing mode, then turn on the deep learning prompts on the image and go through the cases. Such system should be personalized for each radiologist to find how much the computer can trust the gist signal of that individual and find the decision boundaries for the SVM. One of the main challenges in adopting computerized tools for assisting radiologists in reading mammograms is the large number of false positives per image as the tools usually operate at their lax operating points. The false positive annotations may distract the radiologist, possibly cause fatigue^19^, could lead to unnecessary workups^20^, and potentially increase the interpretation time^21^. By using a gist-sensitive deep learning model, we might be able to reduce the false-positive annotations by dynamically changing the operating point of the model.

A major challenge for the application gist signal is inter-radiologist variability. We observed a wide range variability in observers’ performances in the gist experiment. Therefore, the impression of a group of radiologists (i.e. those who performed better at the gist experiment) should not be easily ruled out following the detailed image inspection. This is because the machine model can benefit from the gist responses of these radiologists while the responses from radiologists with poor gist responses could be ignored. None of the reader characteristics predicted their performance in the gist experiment and further studies are required to establish a certain set of criteria for readers, performing better at the gist experiment. Moreover, intra-radiologist’s variability of the gist response is unknown and should be studied in the future before using the signal in clinical decision-making.

The current study had a few limitations. First, cancer prevalence rate in both experiments and benign cases in the second experiment were different from the clinical practice. Secondly, a limited number of cases were included to validate the gist-sensitive deep learning model. The current study was a proof-of-concept study and in future the model should be evaluated on a larger set of radiological images. Moreover, the SVM was personalized for each observer. This means that in practice the model should be trained for each observer before being used. Although personalizing the model makes it more difficult to use compared to non-personalized tools, there is a growing interest in personalized decision-support systems for the radiologists^22^ to account for each radiologist’s unique error making pattern.

In conclusion, the results of both experiments indicated that the gist signal provides malignancy evidence uncorrelated to the information captured by humans or machines following detailed mammogram inspection. The results of the second experiment highlighted the complementary nature of the gist signal to the features captured by a state-of-the-art deep learning model and the usefulness of the gist signal in improving the performance of the model in the breast cancer detection. A potential future work could be exploring the added benefit of pairing high-performing radiologists in the gist experiment with high-performers in the usual viewing condition. This could lead to the enhancement in the double-reading process.

## Materials and methods

All experimental protocols were approved by the Ethics Committee of the University of Sydney (Number: 2019/1017). All methods were carried out in accordance with relevant guidelines and regulations. Informed consent was obtained from all subjects participated in the study. In two separate sets of experiments, we explored if we can find supporting evidence for the hypothesis that the gist signal provides non-overlapping malignancy evidences to the information based on localized image interrogations extracted by (1) radiologists and (2) a deep convolutional neural network. In both experiments, radiological gist signals were collected using the steps described in section "Flashing presentation for collecting the gist signal".

### Flashing presentation for collecting the gist signal

**Figure 5:**
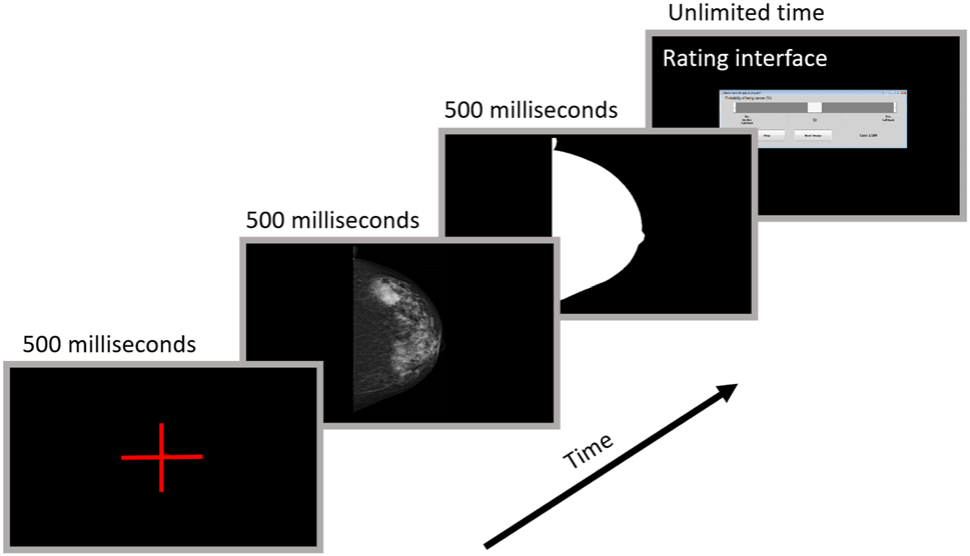
The protocol for presenting the images to collect the gist signal.

The gist response refers to the radiologist’s impression about whether a case contains an abnormality before fixating at any image location. To collect this signal, we used an in-house developed MATLAB application to view images only for 500 ms. Previous studies suggested that 500 ms are enough to capture the gist signal while ensuring that the radiologists were not able to process any area with foveal vision^3,9^. The image presentation protocol to collect the gist signal is shown in Fig. 5. Prior to image presentation, a cross appeared at the center of display and after mammogram presentation, a mask corresponding to the breast area was shown on the display for 0.5 s. Readers were then asked to indicate whether the image contained an abnormality by using a slider to rate their level of confidence from 0 (confident normal) to 100 (confident abnormal).

### Experiment 1: Gist versus normal viewing response

Nineteen radiologists were recruited in 12th General Breast Imaging group meeting of RANZCR. These radiologists were asked to assess a case set of 60 mammographic cases, for which 19 contained biopsy-proven malignancies while the rest of them were normal based on at least two years of follow-up. One of the cases contained two lesions, one in each breast. The cases were selected from the archive of the BreastScreen Australia, in which all cases are independently assessed by two radiologists. All selected cases were missed by one of the two radiologists who assessed the cases. Therefore, all cancer cases are relatively difficult to detect. Table 1 shows the characteristics of cases included in the study.

The radiologists were asked to complete a background questionnaire to provide information about whether they have completed a breast fellowship, whether they work for a screening service, self-reported subspecialty, number of hours spent reading screening mammograms, number of screening cases read per week, and number of years reading mammograms. The characteristics of readers are shown in Table 2.

**Table 2:** Characteristics of cases and radiologists in the first experiment.

Characteristics		Characteristics	Numbers
*Cases*		*Radiologists (out of 19)*	
**Breast density (No/60)**		**Fellowship in breast**	
Almost entirely fatty	13	Yes	7
Scattered areas of fibroglandular density	22	No	12
Heterogeneously dense	12	**Subspecialty**	
Extremely dense	14	Breast	17
**Cancer side (No/20)**		Others	1
Left	**10**	**Number of hours reading mammograms per week**	
Right	10	≤ 10 h	13
**Lesion size**		> 10 h	6
Mean + Std (mm)	10.5 ± 6.1	**Number of cases per week**	
Range (mm)	4.0–26.0	≤ 100 cases	6
**Distance from nipple**		> 100 cases	13
Mean + Std (mm)	60.5 ± 29.2	**Working for a screening service**	
Range (mm)	97.0–119.0	Yes	16
**Cancer type (No/20)**		No	3
Calcification	7	**Number of years reading mammograms**	
Stellate	5	≤ 10 years	8
Discrete Mass	3	> 10 years	11
Architectural Distortion	3		
Non-specific density	2		

Bold values showed the significant *p*-values

Participants were asked to assess the case set, twice. Once in the flashing mode, using the experimental protocol described in section "Flashing presentation for collecting the gist signal" (i.e., gist experiment) and once in the usual viewing and reporting condition (i.e. usual viewing experiment). All participants did the gist experiment prior to the usual viewing experiment. None of them were aware that they were assessing an identical case set in two experiments. In the gist experiment, both right and left crania-caudal images were shown to the radiologists and the maximum of two abnormality scores were assigned to each case.

#### Usual viewing condition

Radiologists used Breast Screen Reader Assessment Strategy (BREAST; http://sydney.edu.au/health-sciences/breastaustralia/) platform ^23,24^ to assess a similar case set in the usual viewing and reporting mechanism. The assessments were done at conference venues in a room, matched with radiologic reporting environments. Readers assessed mammograms on two 5-megapixel reporting monitors and requested to report their findings on the BREAST online platform using the RANZCR rating system^25^, which categorises the findings into five classes: (1) no significant abnormality, (2) benign, (3) equivocal, (4) suspicious, and (5) malignant. No information about the cancer types and prevalence was given to the readers.

#### Statistical analysis

For each case and each radiologist, two scores were available, one from the gist experiment and one from the usual presentation and reporting condition. For each one of the conditions, the empirical ROC curves were generated. To do so, for each unique value of the given scores, the number of false-positives was plotted against the number of true-positives to build the ROC curve. Then, the trapezoidal approximation (empirical method for the AUC calculation) was used to estimate the AUC value. The AUC is scale-invariant as it measures how well predictions are ranked, rather than their absolute values. The bootstrap confidence levels for the AUC values were calculated using the bias-corrected accelerated percentile method^26^ with 100 replicas. Spearman’s correlation coefficient between the AUC values from two viewing conditions was found. The trapezoidal approximation slightly underestimates the AUC value, particularly in the usual viewing condition, where a five-point rating system was used. However, this should not considerably affect the Spearman correlation coefficient as the under-estimation occurs for all radiologists and the trend should still remain the same. In each one of the viewing conditions, the Mann–Whitney U test was used to explore if the reader characteristics resulted in a significant difference between readers’ performances.

For each radiologist and each image type (i.e., cancer or normal), Spearman’s correlation coefficient between the absolute RANZCR score and the gist responses was found. Spearman’s correlation analysis can successfully measure the strength of association between a continuous and an ordinal variable with five or more levels^27^. Although per-radiologist Spearman’s correlation analysis provides interesting insights about the strength of the association between the malignancy signals in these two viewing conditions, it does not leverage the entire power of data and each analysis has a DOF of 58, i.e., 2 subtracted from the total of the number of cases. One possible solution could be pooling the data from all radiologists and then calculate the correlation coefficient. However, as radiologists were assessing the identical set of images, if data from all radiologists had been pooled, each observation would not have been independent, and hence ordinary correlation could not be used to investigate if two ratings were correlated. To handle this dependency, we used the repeated measure correlation (rm-corr), in which the independence within random grouping factors is relaxed^14,15^. Here, the repeated measure correlation estimates the associations between two scores, shared among radiologists. To calculate the repeated measure correlation, as formulated in ^15^, we used the implementation of the measure provided in Pingouin v0.3.3 Python package. The degree of freedom (DOF) for calculating the repeated measure correlation^15^ was N × (k−1)^−1^, where N shows the number of radiologists and k shows the number of measurements available for each radiologist. For the cancer category, the DOF was 341 while it was 759 for the normal category. When two categories were combined, the DOF was 1120. Therefore, it should be noted that DOF was higher for the normal category because of its higher sample size. The absolute gist and RANZCR scores were used for calculating the rm-corr. The rm-corr coefficient, similar to the Pearson correlation, provides information on the strength and direction of the linear relationship between two variables, rather than estimating a parameter in a linear equation that can be used to predict RANZCR scores from the gist responses. Therefore, rm-corr coefficient remains identical, when scores from a grading system were scaled. In addition, to compute overall correlation, as measured by the repeated measure correlation, we also calculated Spearman’s correlation for each radiologist. All correlation values were separately calculated for normal and cancer cases.

Using the mixed linear regression model^16^, which handles within subject dependencies, we investigated if the gist response, lesion size (in mm), lesion type (calcification or not), and mammographic density were predictors of radiologists’ RANZCR ratings in the usual viewing condition. To build the model and get coefficients for each predictor, statsmodels v0.11.1 Python package was utilized.

We also compared the radiologist’s performances in the two viewing conditions. Two ROC curves for each reader were generated and the AUC values was calculated. The Spearman correlation between the AUC values of the gist experiment and AUC values from the usual reporting and presentation condition was also calculated. We also built a linear regression model to relate the AUC in the gist experiment to that derived from the usual reporting condition and investigated if the regression coefficient was significantly different from zero.

### Experiment 2: Gist to complement to a deep learning model’s diagnosis

#### Dataset

To fulfill the second aim, we recruited 53 radiologists at the Radiological Society of North America (RSNA) as well as RANZCR annual meetings and asked radiologists to evaluate a case set of 80 images in the gist experiment. The case set contained 20 normal, 40 benign, and 20 cancer cases. All cancer cases were biopsy-proven malignancies. Characteristics of these cases are shown in Table 3. Normal cases were those that remained normal at least based on a two-year follow-up. Here, in addition to the normal cases, we included the benign cases as for using such tool in the clinical practice, it should be able to help with the challenging cases.

**Table 3:** Characteristics of cancer cases in the second experiment.

Type		Location		Lesion size (mm)	
Architectural Distortion	2	Central	5	Mean	11.35
Calcification	1	Lower Inner (inferior medial)	2	Std	5.26
Discrete Mass	2	Lower Outer (inferior lateral)	1	Min	5
Non-specific density	6	Retro Areolar	1	Max	24
Spiculated Mass	3	Upper Inner (superior medial)	1		
Stellate	6	Upper Outer (superior lateral)	10		

Benign cases were selected from the BREAST^24^ archive based on the opinion of a senior radiologist. Since 2011, more than 1000 readers, have used the BREAST platform for reading nine case sets of screening images. Hence, for each case, many radiological reports were available. We retrieved all non-malignant cases, frequently annotated as 2 (benign findings) or 3 (indeterminate/equivocal findings) by most radiologists in BREAST platform. Based on opinion of a senior radiologist with 30+ years of experience in reading screening mammograms, we selected a subset of 20 images from these cases, representing BI-RADS 2. We also searched in the BREAST archive for cases annotated as 3 or above by at least at least one-third of radiologists who assessed them. With the help of the experienced radiologist, we reviewed these cases and selected 20 images with confirmed benign findings. Hence two sets of benign of images, B1 (the first set) and B2 (the second set), were included.

In the previous experiment all recruited readers had an interest in breast imaging as the main aim of the study was investigating how the gist signal is related to the radiologists’ ratings in usual presentation condition. However, in the current experiment, the primary aim was exploring if the gist signal provided any complementary information to the decision made by a computer. Therefore, any interested radiologist was eligible to participate in the study.

#### Combining radiological gist with the deep learning model’s output

A state-of-the-art deep learning-based tool^13^ for breast cancer detection was adopted. Briefly, the network has two components. The first part is a convolutional neural network, which was fine-tuned using 1903 input images to categorize images patches into five classes, representing benign calcifications, malignant classification, benign masses, malignant masses, and background. The patch classifier has a VGG16 architecture and an input size of 224 × 224 pixels. To handle inputs of any size, the last non-convolutional layers of the patch classifier were omitted, and a “non-linear deep filter” was built to scan the entire image for cancer cues. In summary, for building the patch classifier, Shen et al.^13^ compared the VGG network^28^ and the residual network (ResNet)^29^. As the VGG structure outperformed the ResNet, here we used the VGG architecture as the patch classifier. The VGG16 consists of five convolutional VGG blocks followed by two fully connected layers. Each VGG block One VGG block is a sequence of convolutional layers, followed by a maximum pooling layer for spatial down-sampling. Shen et al. modified the original VGG16 and replaced the fully connected layers with a global average pooling layer to compute the average activation of each feature map for the output of the last VGG block. To train the patch classifier, a three-stage strategy was taken. First, with a learning rate of 10^−3^, the last layer of a network, previously trained on the ImageNet dataset, was trained for 3 epochs while teh rest of the layers were frozen. This was done as the bottom layers represent primitive features, which should be preserved across different tasks, and training these layers with a large learning rate may destroy the features already learned by this layer. In the next stage of training, the top 11 layers were trained using a learning rate of 10^−4^ for 10 epochs. Finally, in the last stage, all layers were trained using a learning rate of 10^−5^ for 37 epochs. Therefore, the network was trained for 50 epochs in total.

To perform classification on large images or segment a large image such as histopathological images^30^, using a classifier in a sliding window fashion to classify or segment local image patches to generate a grid of outputs is a common approach. Such a strategy requires optimizing weights of a patch classifier and also a method to summarize the grid of outputs to provide the final image-level classification result. To combine these two steps in order to train the network on the whole mammograms, Shen et al.^13^ first trained the patch classifier. Inputting a single image with a size of 224 × 224 pixels to the patch classifier resulted in a single probabilistic output of five classes, representing benign calcification, malignant calcification, benign mass, malignant mass, and background. After sweeping the entire image with the patch classifier, we have a u × v grid of probabilistic outputs of five classes or five “heatmaps”. The values for u and v depend on the stride of the patch classifier and the total image size. Therefore, we can assume a deep filter, i.e., the patch classifier, sweeps the entire image looking for “suspicious areas” and outputs five heatmaps.

More convolutional layers can be added on the top of the heatmaps to transform the patch classifier to the final image-level classifier. Adding a convolutional layer on the top of the patch classifier would turn the patch classifier to a deep filter, which is effectively being utilized by the top layer to scan the image. Using functions *T* and *P* to represent the top layers and the patch classifier, the whole image classifier, *H*, can be written as H(i) = T(P(p)), where *i* and *p* represent the input mammogram and the extracted patch. Here the heatmaps were fed into two additional convolutional blocks followed by pooling, flattening, and fully connected layers (i.e., function *T*) to categorize the entire image as normal and abnormal.

An important implication of the above-mentioned formulation is possibility of an “end-to-end” training of the network on a dataset with image-level labels rather than detailed annotations of image regions. After training the patch classifier, to conduct the end-to-end training, a two-stage strategy was taken to first train the top layers corresponding to function *T* with a learning rate of 10^−4^ and then train all layers, or function *H*, with a learning rate of 10^−5^. Similar to the strategy used for training the patch classifier, the two-stage end-to-end training approach ensures that large the learning rate does not destroy the features, already learned by the patch classifier. The model achieved an AUC of 0.95 for categorizing a set of images from the INbreast database^31^.

We used a data set of 4500 screening cases retrieved from Breast Screen Australia to further fine-tune the model. Our dataset contained 3, 494 normal cases and 1, 006 biopsy-proven cancer cases, and four images were available for each case. For normal cases, we used images of both breasts, but for cancer cases we included the cancer-containing side. The fine-tuning was done in a University of Sydney’s High-Performance Computing facility equipped with Nvidia V100 GPUs. We used Adam as the optimizer with a learning rate of 10^−5^ and weight decay of 0.01. We originally set number of epochs to 200 as per suggested in the original paper, but as we observed improvements in the performance, we continued the training for a total of 300 epochs.

None of the 80 cases, used in the gist experiment were used during training of the model. After completing the training step, we fed all four available images for 80 cases to the model. For each side, we took the average of CC and MLO views. To get the patient-level abnormality score, we considered the maximum abnormality score of left and right sides.

In order to combine the abnormality score from the network for a case with the gist response, both values were fed into a support vector machine (SVM). Tuning the hyper-parameter of the SVM is essential for achieving the highest performance. We utilized the Bayesian optimization^32^ with expected improvement per second + as its acquisition function, which select points that are not only likely to yield high accuracy, but that are also likely to be evaluated quickly^33^. The plus (+) edition of the expected improvement per second has a property which enables it to escape from a local optimum and returns to exploratory behavior when a region is likely to be over-exploited.

The SVM was personalized for each reader and the performances were evaluated using leave-one-out cross-validation (LOOCV). The model was also built for the average reader, whose gist response for each image was obtained by averaging the abnormality scores given by all 53 readers to that image. For all readers and the average reader, the kernel function was set to the polynomial with order of 4 and box constraints and kernel scale was optimized using the Bayesian optimization.

## Data Availability

The data that support the findings of this study are available from the corresponding author upon reasonable request.
